# Minichromosome maintenance protein 2 and 3 promote osteosarcoma progression via DHX9 and predict poor patient prognosis

**DOI:** 10.18632/oncotarget.15474

**Published:** 2017-02-18

**Authors:** Dong-dong Cheng, Hui-zhen Zhang, Jun-qing Yuan, Shi-jie Li, Qing-cheng Yang, Cun-yi Fan

**Affiliations:** ^1^ Department of Orthopedics, Shanghai Jiao Tong University Affiliated Sixth People's Hospital, Shanghai, 200233, China; ^2^ Department of Pathology, Shanghai Jiao Tong University Affiliated Sixth People's Hospital, Shanghai, 200233, China

**Keywords:** MCM2, MCM3, DHX9, proliferation, osteosarcoma

## Abstract

A label free quantitative proteomic approach (SWATH™ experiment) was performed to identify tumor-associated nuclear proteins that are differentially expressed between osteosarcoma cells and osteoblast cells. By functional screening, minichromosome maintenance protein 2 (MCM2) and minichromosome maintenance protein 3 (MCM3) were found to be related to osteosarcoma cell growth. Here, we show that knockdown of MCM2 or MCM3 inhibits osteosarcoma growth *in vitro* and *in vivo*. In co-immunoprecipitation and co-localization experiments, MCM2 and MCM3 were found to interact with DExH-box helicase 9 (DHX9) in osteosarcoma cells. A rescue study showed that the decreased growth of osteosarcoma cells by MCM2 or MCM3 knockdown was reversed by DHX9 overexpression, indicating that MCM2 and MCM3 activity was DHX9-dependent. In addition, the depletion of DHX9 hindered osteosarcoma cell proliferation. Notably, MCM2 and MCM3 expression levels were positively correlated with the DHX9 expression level in tumor samples and were associated with a poor prognosis in patients with osteosarcoma. Taken together, these results suggest that the MCM2/MCM3–DHX9 axis has an important role in osteosarcoma progression.

## INTRODUCTION

Osteosarcoma, a malignant tumor of mesenchymal origin, is the most common primary bone sarcoma in children and adolescents [1]. Improvements in surgical treatment and the development of new chemotherapy drugs have significantly decreased the number of amputations. Over time, patient survival has improved from less than 30% to greater than 70% [2]. Despite these advances, the survival rates for osteosarcoma have reached a plateau [3]. Nowadays, many patients remain insensitive to chemotherapy and have a poor prognosis. Therefore, there is still an urgent need to investigate novel chemotherapeutic targets.

Accumulating evidence indicates that nuclear proteins, located in the nuclei of cells, play important roles in the genesis and progression of various cancers. Shahbazi *et al*. [4] reported that the repression of the nuclear protein TP53INP1 is an important co-factor for N-Myc oncogenesis, suggesting a new therapeutic approach for N-Myc-induced neuroblastoma. MTHFD2, a nuclear protein, co-localizes with DNA replication sites and sufficiently promotes cancer cell proliferation [5]. Pedrola *et al*. reported that Nuclear Protein 1 (NUPR1) was a direct target of the transcription factor ETV5 in endometrial cancer; the inhibition of NUPR1 reduced cell migration and invasion *in vitro* and reduced tumor growth and dissemination in an orthotopic endometrial cancer model [6]. Mass spectrometry-based proteomics is a powerful tool for biological and clinical research and enables relatively comprehensive global analyses [7, 8]. Relative quantitation with stable isotope labeling or label-free methods is widely used to study differential protein expression profiles [9–11]. SWATH™ is a recently developed label-free quantitative proteomic approach based on a data-independent acquisition (DIA) strategy and it can successfully quantify compounds with high complexity [12, 13]. The systematic analysis of the expression levels of nuclear proteins using SWATH™ provides an effective approach to identify molecules involved in carcinogenesis.

In the present study, a SWATH™ analysis was performed using osteosarcoma cell lines (MNNG/HOS and U2OS) and an osteoblast cell line (hFOB 1.19). By functional screening, MCM2 and MCM3, components of the MCM2-7 complex, were found to be related to osteosarcoma proliferation. Several studies have demonstrated that MCM proteins can be used as proliferation markers to predict the behaviors of diverse tumors [14, 15]. However, the functions and molecular mechanisms of MCM2 and MCM3 in osteosarcoma are still far from fully understood. The results of this study demonstrated that knockdown of MCM2 or MCM3 inhibited osteosarcoma cell growth *in vitro* and *in vivo*. MCM2 and MCM3 were found to interact and co-localize with DHX9 in osteosarcoma. Overexpression of DHX9 could rescue MCM2 or MCM3 knockdown-induced tumor inhibition. Further analyses showed that the inhibition of DHX9 hindered osteosarcoma cell proliferation. Notably, we found that MCM2 and MCM3 were independent prognostic factors for tumor-free survival (TFS) and overall survival (OS) in patients with osteosarcoma. A positive correlation between MCM2 or MCM3 and DHX9 expression was detected in osteosarcoma tissues. Taken together, MCM2 and MCM3 promote osteosarcoma progression via associations with DHX9 and thus are potential therapeutic targets for patients with osteosarcoma.

## RESULTS

### Comparative nuclear proteome profile of osteoblast cells versus osteosarcoma cells

**Figure 1:**
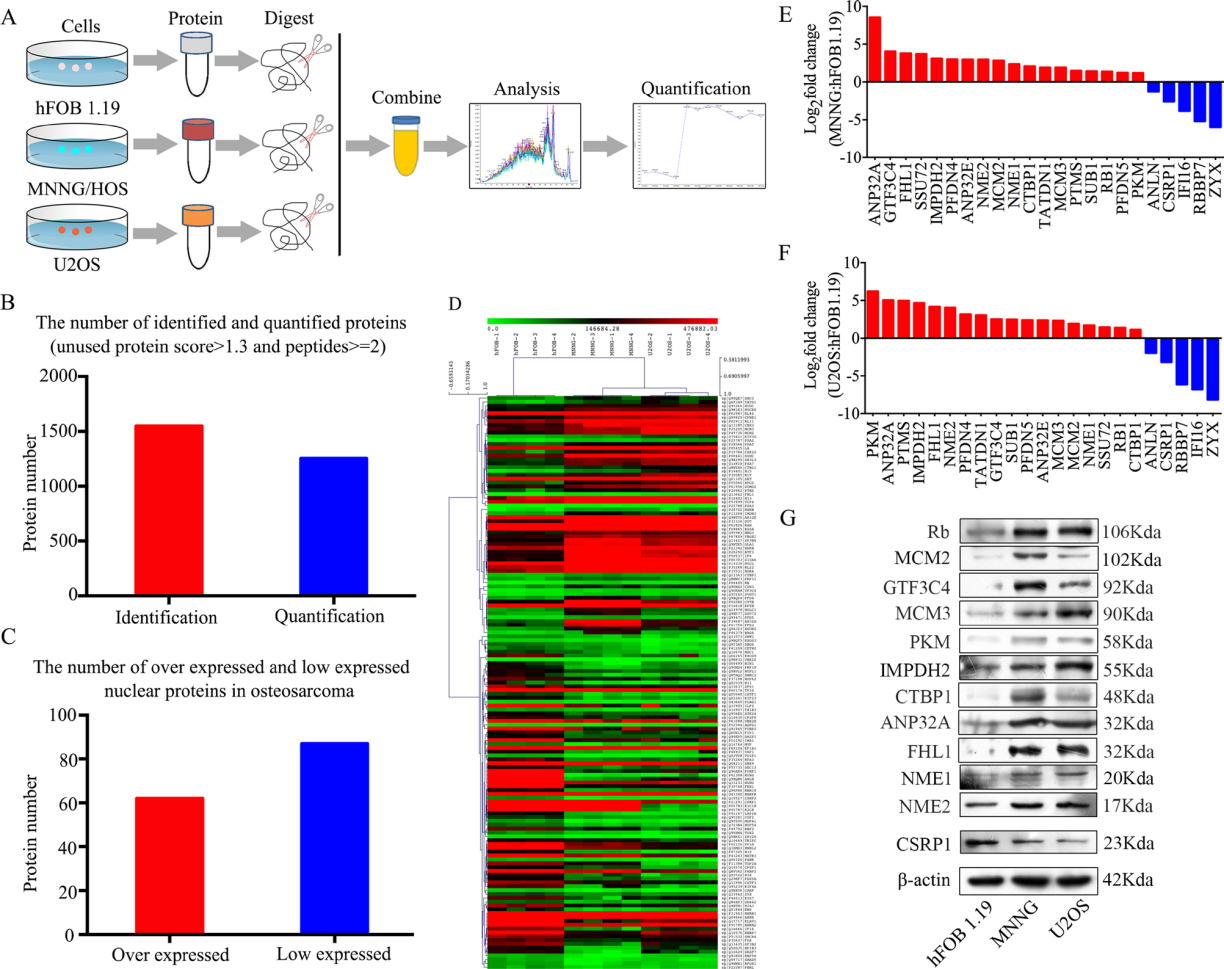
Experimental workflow for the SWATH™ quantitative proteomics analysis (**A**) Flowchart of the proteomic analysis. (**B**) Numbers of identified and quantified nuclear proteins in the SWATH™ analysis. (**C**) Numbers of upregulated and downregulated nuclear proteins in osteosarcoma cells. (**D**) A heat map of dysregulated proteins in osteosarcoma. (**E**, **F**) The mRNA expression levels of 18 upregulated and 5 downregulated nuclear proteins in MNNG/HOS and U2OS cells compared with hFOB 1.19 cells were verified by qRT-PCR. (**G**) Representative blots display the protein expression levels of 11 upregulated and one downregulated nuclear proteins. β-actin was used as an internal control.

In the present study, SWATH™ measurement was used to determine differentially expressed nuclear proteins between osteoblast cells (hFOB 1.19) and osteosarcoma cells (MNNG/HOS and U2OS). The flowchart of the proteomic analysis is shown in Figure 1A. In the SWATH™ experiments, a total of 1549 proteins (global FDR < 1%) were identified. To ensure confident quantitative results, we selected the proteins with at least 2 peptides identified using PeakView^®^ for subsequent analyses. Using this filter, 1254 proteins were quantified repeatedly in four technical replicates (Figure 1B). In total, 62 upregulated proteins and 87 downregulated proteins were identified in all osteosarcoma cell lines compared to the osteoblast cell line (Figure 1C). A heatmap of deregulated proteins in osteosarcoma cells is shown in Figure 1D. Gene ontology (GO) and Kyoto Encyclopedia of Genes and Genomes (KEGG) pathway analyses [16] were performed to evaluate the differentially expressed proteins (Supplementary Figure 1). 18 upregulated proteins and 5 downregulated proteins were selected to further validate the proteomic results. The mRNA expression levels of these proteins, as determined by qRT-PCR, were consistent with the results of the proteomic analysis (Figure 1E and 1F). Then, 11 upregulated proteins, i.e., Rb, MCM2, GTF3C4, MCM3, PKM, IMPDH2, CTBP1, ANP32A, FHL1, NME1, and NME2, and one downregulated protein, CSRP1, were chosen to verify the results at the protein level. The expression levels of these proteins were consistent with the results of the proteomic analysis (Figure 1G). These results collectively support the feasibility of our approach for identifying osteosarcoma-associated nuclear proteins.

### Functional screening osteosarcoma proliferation-related nuclear proteins

**Figure 2:**
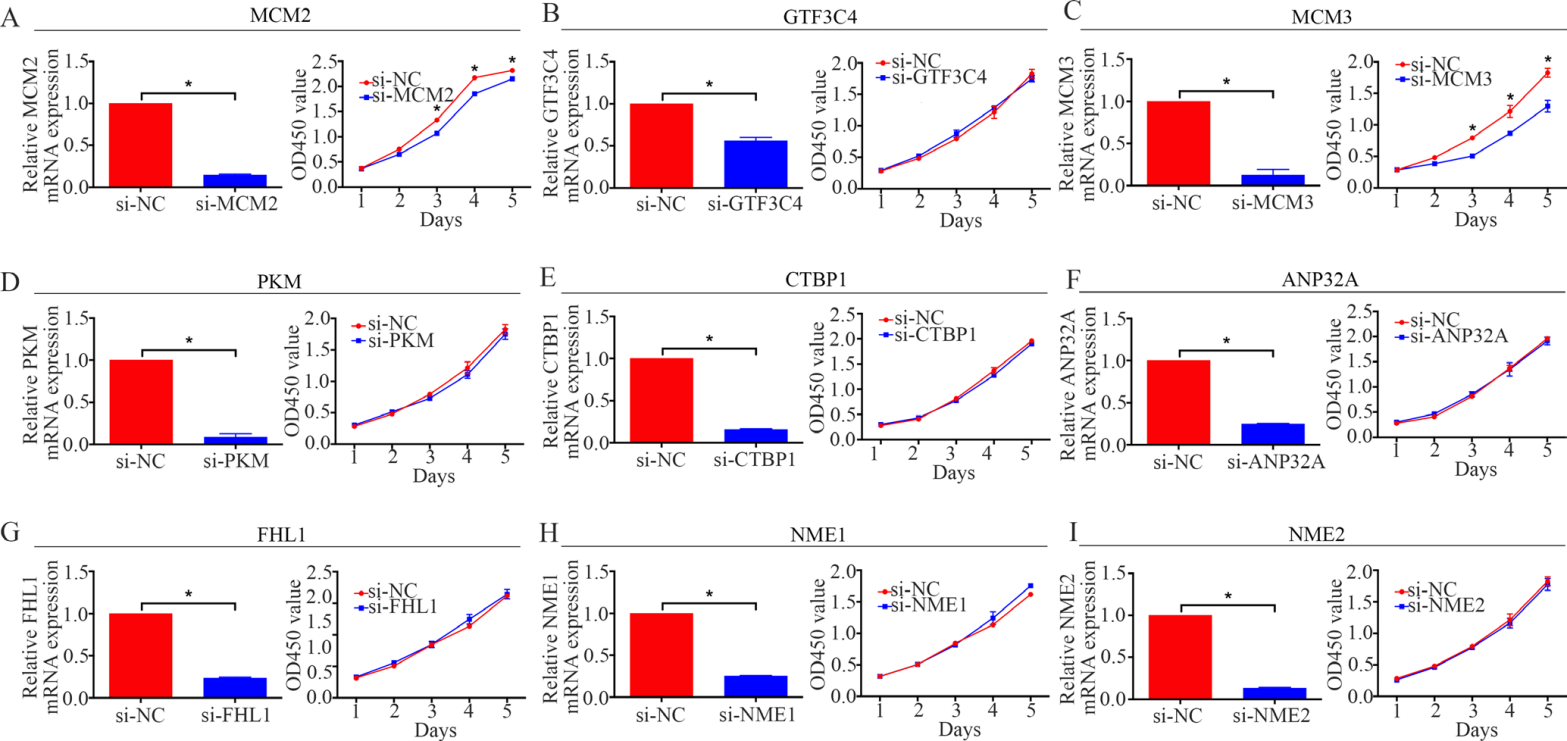
Functional screening of 9 candidate nuclear proteins in the MNNG/HOS cell line A CCK-8 assay was used to detect the proliferation of MNNG/HOS cells after transfection with siRNA. *P < 0.05.

To determine osteosarcoma proliferation-related nuclear proteins, functional screening by siRNA knockdown and CCK-8 assay [17] were performed. We chose 9 nuclear proteins, namely, MCM2, GTF3C4, MCM3, PKM, CTBP1, ANP32A, FHL1, NME1, and NME2, which might be associated with osteosarcoma oncogenesis. The results showed that the inhibition of MCM2 or MCM3 could restrain the proliferation of osteosarcoma cells, whereas the inhibition the other proteins had no effect on the proliferation of osteosarcoma cells (Figure 2). In sum, we hypothesized that MCM2 and MCM3 were associated with the proliferation of osteosarcoma cells.

### The role of MCM2 and MCM3 in osteosarcoma cells *in vitro*

**Figure 3:**
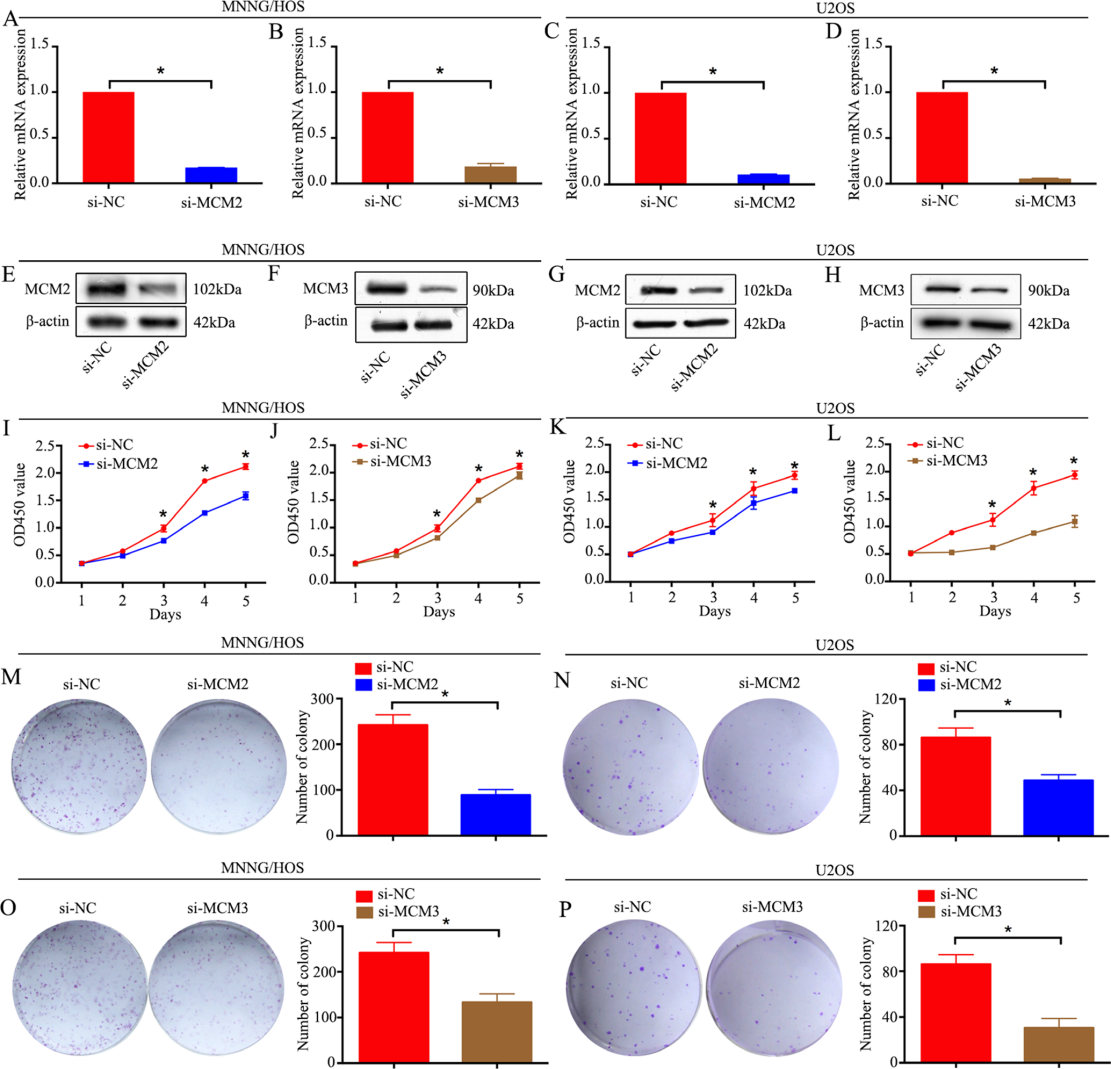
Knockdown of MCM2 or MCM3 inhibits osteosarcoma cell proliferation *in vitro*. (**A**–**H**) The mRNA and protein expression levels were validated after MCM2-specific or MCM3-specific siRNA transfection by qRT-PCR and western blotting in MNNG/HOS cells and U2OS cells. (**I**–**L**) CCK-8 assays were performed after siRNA transfection in MNNG/HOS cells and U2OS cells. (**M**–**P**) Colony formation assay for MCM2-silenced or MCM3-silenced osteosarcoma cells and control cells. Data are representative of results from three independent experiments. *P < 0.05.

To explore the functional significance of MCM2 and MCM3 in osteosarcoma, MCM2-specific or MCM3-specific siRNA was used to knockdown MCM2 or MCM3 in two osteosarcoma cell lines, MNNG/HOS and U2OS. MCM2 or MCM3 knockdown was validated by qRT-PCR and western blot analyses. The mRNA and protein expression levels of MCM2 and MCM3 were significantly reduced after transfection with siRNA in MNNG/HOS and U2OS cells (Figure 3A–3H). A CCK-8 assay was used to detect cell proliferation. The data revealed that the knockdown of MCM2 or MCM3 significantly inhibited the growth of osteosarcoma cells (Figure 3I–3L). Similarly, a colony formation assay showed that the knockdown of MCM2 or MCM3 attenuated the formation of cell colonies (Figure 3M–3P). These results suggest that the overexpression of MCM2 or MCM3 has an oncogenic role in osteosarcoma.

### Knockdown of MCM2 or MCM3 inhibits tumor growth *in vivo*

**Figure 4:**
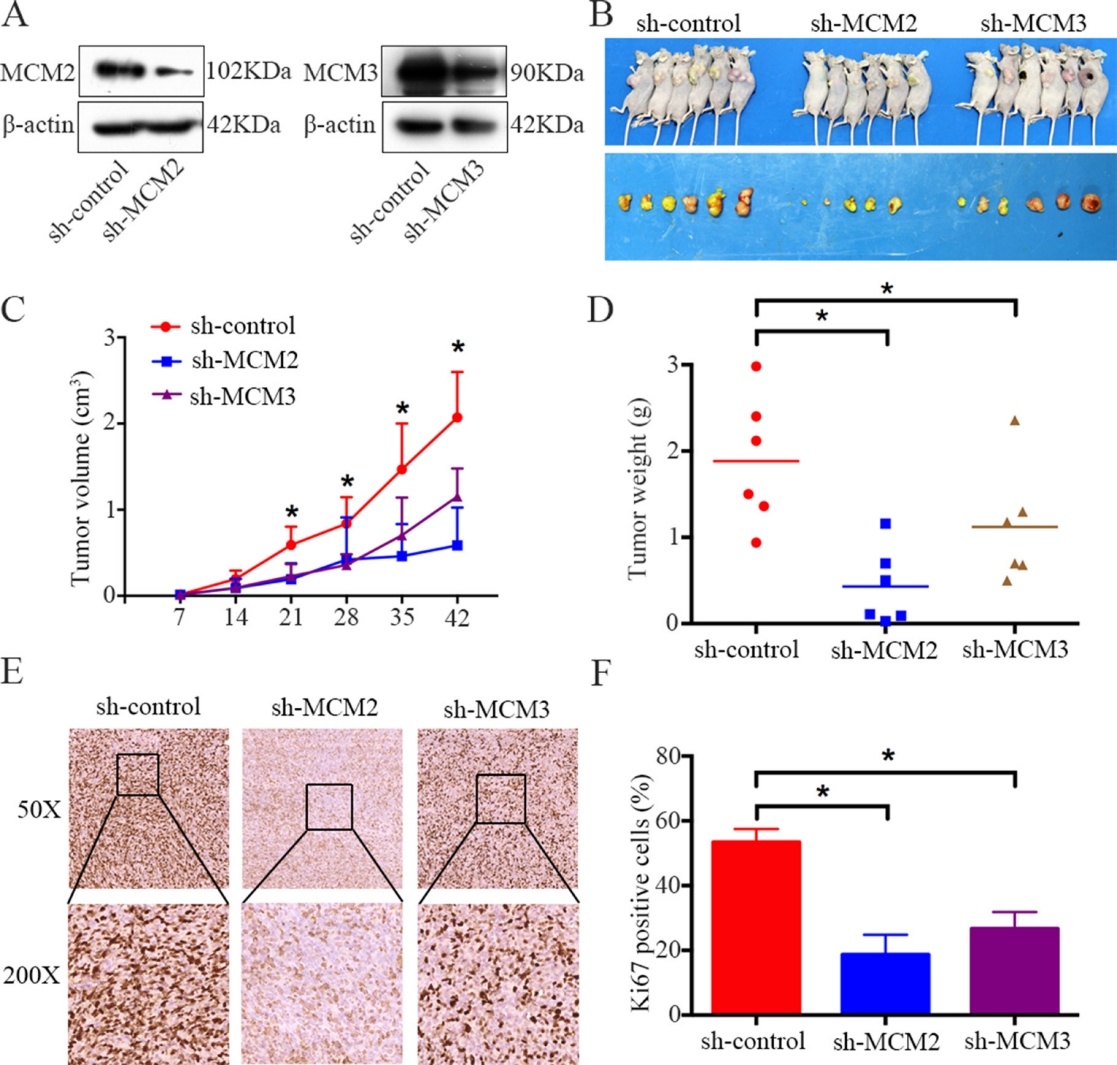
Knockdown of MCM2 or MCM3 inhibits osteosarcoma cell growth *in vivo* (**A**) Representative blots display the protein expression of MCM2 and MCM3 in MNNG/HOS cells stably expressing sh-MCM2 or sh-MCM3. β-actin was used as an internal control. (**B**) The upper photograph shows tumor-bearing mice and the lower photograph shows tumors when mice were euthanized. (**C**) Growth curve drawn by measuring tumor volumes on the indicated days. (**D**) The diagram shows tumor weights in the sh-control, sh-MCM2, and sh-MCM3 groups. (**E**, **F**) Representative images of ki-67 staining in the sh-control, sh-MCM2, and sh-MCM3 groups. Magnification, ×50, ×200. *P < 0.05.

To further determine the effects of MCM2 and MCM3 on osteosarcoma growth *in vivo*, MNNG/HOS cells stably expressing sh-control, sh-MCM2, and sh-MCM3 were constructed. Protein expression was validated by western blotting (Figure 4A). Then, cells were subcutaneously injected into the left scapulas of nude mice, and the animals were closely monitored for tumor growth for 6 weeks. The tumor growth curve demonstrated that the knockdown of MCM2 or MCM3 significantly inhibited tumor growth *in vivo*. The tumor volume and weight were smaller in the MCM2 and MCM3 knockdown groups than the scramble group (Figure 4B–4D). As shown in Figure 4E and 4F, based on an immunohistochemical analysis, the levels of the tissue proliferation marker Ki-67 [18] was lower in the sh-MCM2 and sh-MCM3 tumors compared to those in sh-control tumors. Taken together, these results indicated that MCM2 or MCM3 knockdown could hinder the tumorigenesis of osteosarcoma cells *in vivo*.

### MCM2 and MCM3 interact with DHX9 in osteosarcoma cells

**Figure 5:**
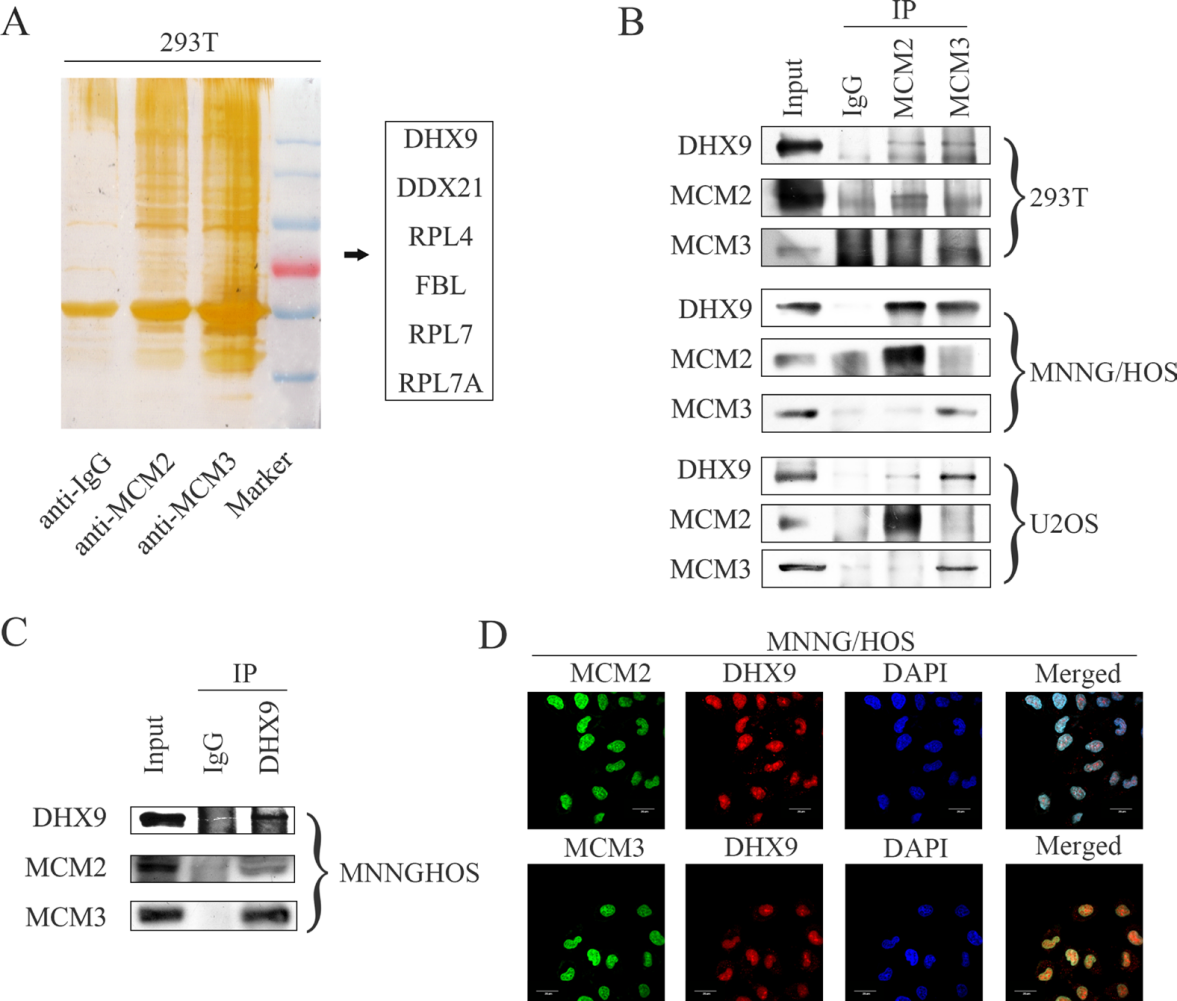
MCM2 and MCM3 interact with DHX9 in osteosarcoma cells (**A**) The silver staining for proteins separated by SDS-PAGE after IgG, MCM2, or MCM3 pull-down in 293T cells. (**B**) Whole-cell lysates were immunoprecipitated with the anti-MCM2 antibody or anti-MCM3 antibody followed by immunoblotting with anti-MCM2, MCM3, and DHX9 antibodies in the 293T cell line and the indicated osteosarcoma cell lines. IgG was used as a negative control. (**C)** Whole-cell lysates were immunoprecipitated with the anti-DHX9 antibody followed by immunoblotting with anti-MCM2, MCM3, and DHX9 antibodies in the MNNG/HOS cell line. IgG was used as a negative control. (**D**) An immunofluorescence study was performed using anti-MCM2, MCM3, and DHX9 antibodies in the MNNG/HOS cell line. DAPI was used as a control for nuclear staining.

In order to determine the underlying mechanism by which MCM2 and MCM3 promote osteosarcoma, co-immunoprecipitation was performed in 293T cells. After protein identification by mass spectrometry, 6 proteins (DHX9, DDX21, RPL4, FBL, RPL7, and RPL7A) were chosen for further verification (Figure 5A). To assess interactions between proteins, we pulled down MCM2 or MCM3 and probed their interactions with other proteins in 293T cells and osteosarcoma cells. We found that MCM2 and MCM3 specifically interacted with DHX9, as indicated by an immunoprecipitation assay (Figure 5B), whereas no interactions were observed with other proteins (Supplementary Figure 2). No interaction was found between MCM2 and MCM3 in osteosarcoma cells. In addition, DHX9 interacted with MCM2 and MCM3 in MNNG/HOS cells (Figure 5C). We also confirmed the co-localization of MCM2 and MCM3 with DHX9 in MNNG/HOS cells by confocal microscopy (Figure 5D). Thus, MCM2 and MCM3 are associated with DHX9 in osteosarcoma cells.

### Knockdown of MCM2 or MCM3 inhibits osteosarcoma cell proliferation via DHX9

**Figure 6:**
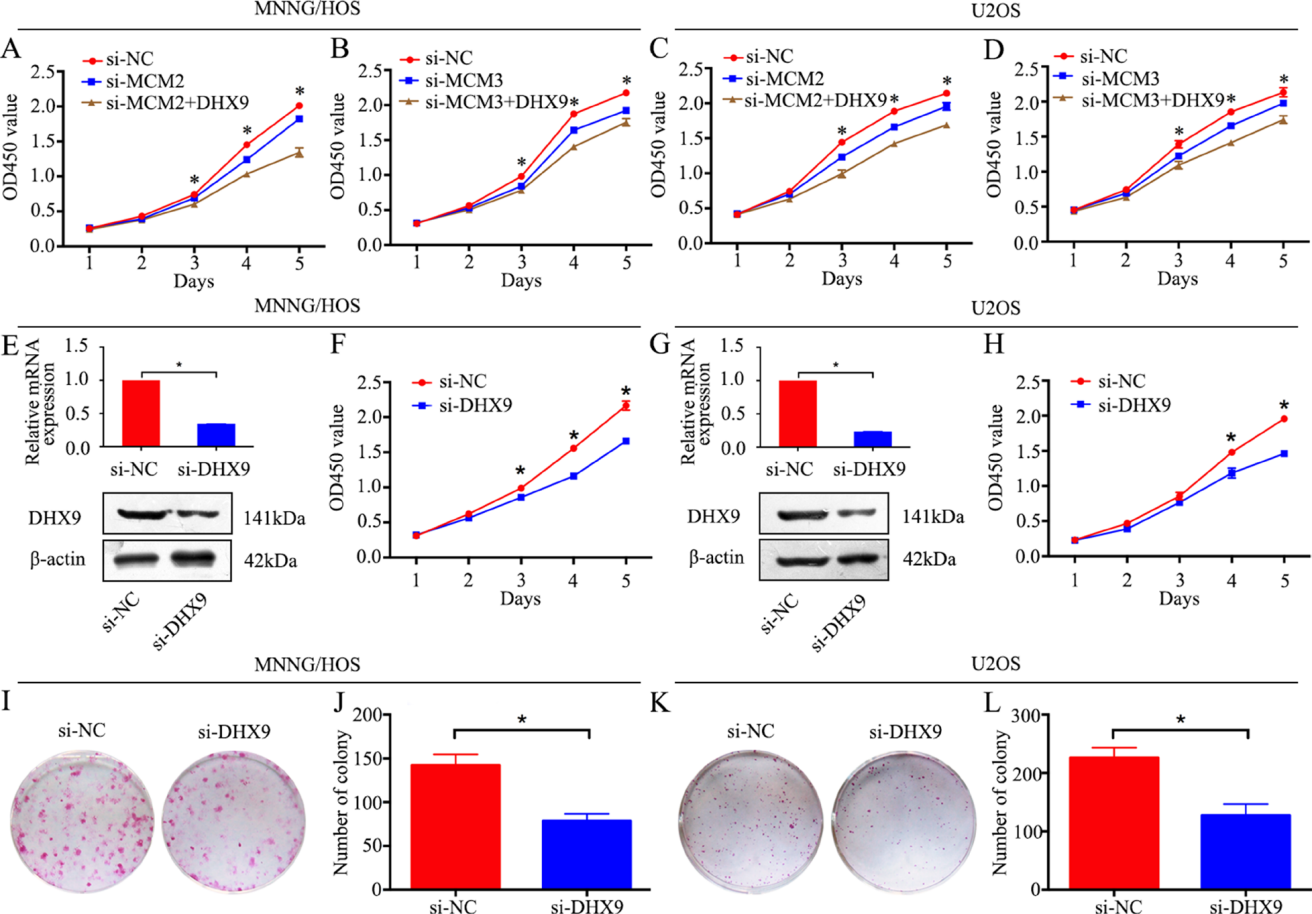
Knockdown of MCM2 or MCM3 inhibits osteosarcoma cell proliferation via DHX9 (**A**, **C**) A CCK-8 assay was used to detect tumor cell proliferation after transfection with pcDNA 3.1-DHX9 plus si-MCM2 or si-NC. (**B**, **D**) CCK-8 assay was used to detect tumor cell proliferation after transfection with pcDNA 3.1-DHX9 plus si-MCM3 or si-NC. (**E**, **G**) The mRNA and protein expression levels were validated after DHX9-specific siRNA transfection by qRT-PCR and western blotting in MNNG/HOS and U2OS cells. (**F**, **H**) A CCK-8 assay was used to detect tumor cell proliferation after transfection with si-DHX9 in MNNG/HOS and U2OS cells. (**I**, **J**, **K**, **L**) Colony formation assay for DHX9-silenced osteosarcoma cells and control cells. Data are representative of three independent experiments. *P < 0.05.

Our data demonstrated that knockdown of MCM2 or MCM3 inhibited osteosarcoma cell proliferation. However, the role of DHX9 in osteosarcoma and whether MCM2 and MCM3 function via DHX9 are largely unknown. Since MMC2 and MCM3 interact with DHX9 in osteosarcoma cells, it is plausible that they function via DHX9, and DHX9 plays an important role in osteosarcoma cell proliferation. We overexpressed DHX9 in MCM2 or MCM3 knockdown MNNG/HOS and U2OS cells. As expected, DHX9 overexpression rescued the MCM2 or MCM3 knockdown-induced cell growth inhibition (Figure 6A–6D). Then, DHX9-specific siRNA was used to knockdown DHX9 in osteosarcoma cells. DHX9 knockdown was validated by qRT-PCR and western blotting (Figure 6E and 6G). A CCK-8 assay and colony formation assay were used to detect cell proliferation. These data revealed that the knockdown of DHX9 significantly inhibited the growth of osteosarcoma cells (Figure 6F, 6H, and 6I–6L). Collectively, these results reveal that knockdown of MCM2 or MCM3 inhibits osteosarcoma cell proliferation via DHX9 and DHX9 plays an oncogenic role in osteosarcoma.

### High expression of MCM2 and MCM3 is significantly associated with worse prognosis in osteosarcoma

**Figure 7:**
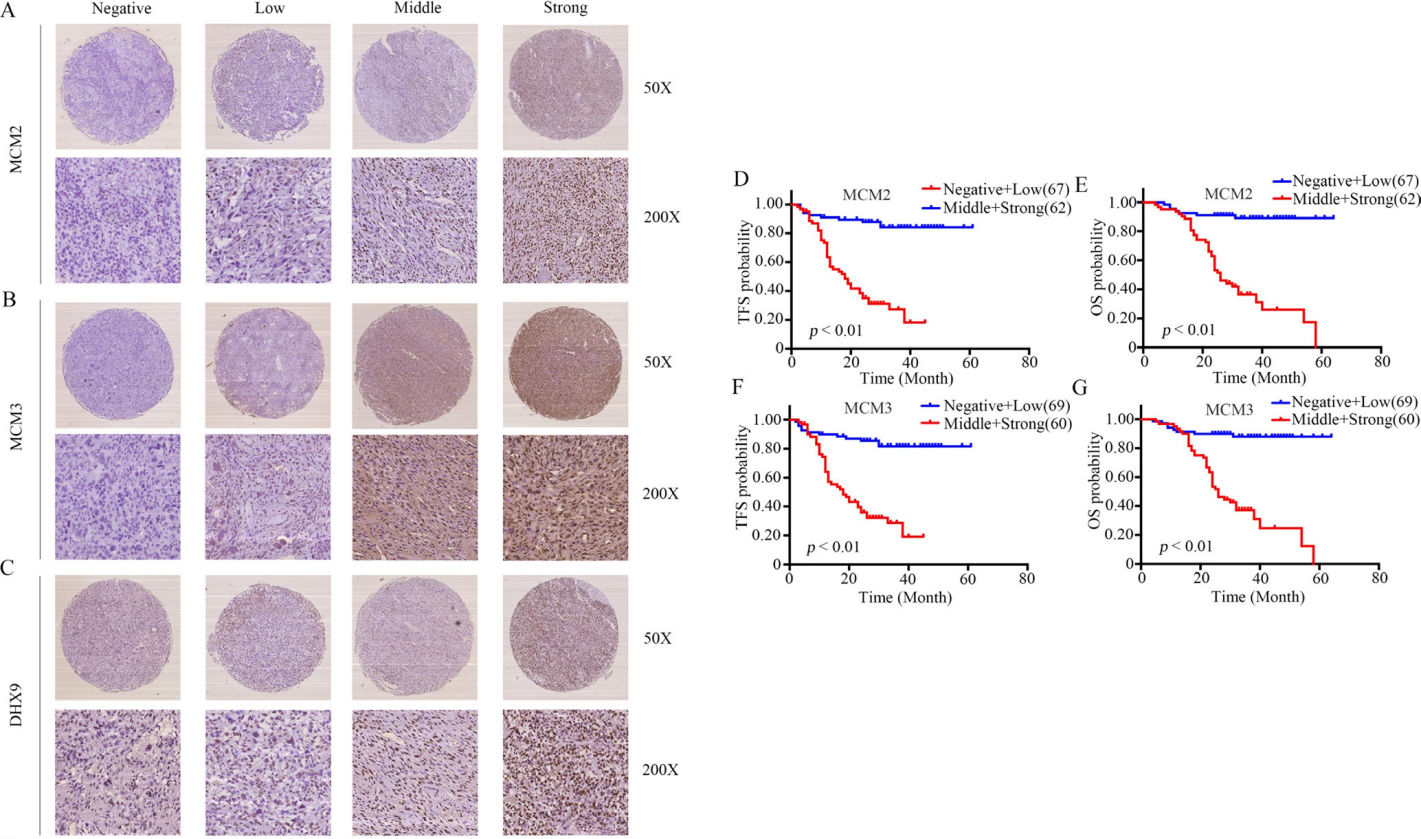
Clinical significance of MCM2 and MCM3 in osteosarcoma patients (**A**–**C**) Representative IHC images of the expression levels (negative: score = 0, low: score = 1, middle: score = 2, and strong: score = 3) of MCM2, MCM3, and DHX9 in osteosarcoma tissues. Original magnification: 50×, 200×. (**D**–**G**) The impact of MCM2 and MCM3 on TFS and OS for patients with osteosarcoma.

To further determine the clinicopathological significance of MCM2 and MCM3 in osteosarcoma, we performed an immunohistochemical (IHC) analysis of MCM2 and MCM3 using a tissue microarray that included an independent set of 129 cases of osteosarcoma. Representative images of MCM2 and MCM3 expression results are shown in Figure 7A and 7B. The correlations between MCM2, MCM3 expression levels and the clinicopathological characteristics of osteosarcoma patients are summarized in Table 1. The expression levels of MCM2 and MCM3 were higher in patients with clinically advanced Enneking stage than those at an early stage (*P* = 0.000). Further analysis indicated that MCM2 and MCM3 levels were positively correlated with recurrence (*P* = 0.000), indicating that MCM2 and MCM3 play important roles in osteosarcoma recurrence. A univariate analysis showed that TFS was related to MCM2 (*P* = 0.000), MCM3 (*P* = 0.000), and Enneking stage (*P* = 0.000) (Table 2). OS was related to MCM2 (*P* = 0.000), MCM3 (*P* = 0.000), and Enneking stage (*P* = 0.000) (Table 2). Variables that exhibited significant differences in a univariate analysis were included in a multivariate analysis. A multivariate Cox regression analysis showed that MCM2 (χ^2^ = 4.333, HR = 1.603, *P* = 0.037) and MCM3 (χ^2^ = 4.939, HR = 1.696, *P* = 0.026) were independent prognostic factors for TFS (Table 3). The Cox proportional hazards model showed that MCM2 (χ^2^ = 4.568, HR = 1.690, *P* = 0.033) and MCM3 (χ^2^ = 4.757, HR = 1.718, *P* = 0.029) were independent prognostic factors for OS (Table 3). The OS and DFS curves for this cohort are presented in Figure 7D–7G. All other factors, including gender, age, tumor location, tumor necrosis rate, cortical destruction, and Enneking stage did not have any significant influence on prognosis. We also performed Kaplan–Meier survival analyses using microarray data (http://www.kmplot.com) obtained from breast and lung cancer patients. We found that MCM2 and MCM3 expression were negatively correlated with patient OS in breast cancer and lung cancer (Supplementary Figure 3). Finally, we detected the expression of DHX9 in osteosarcoma tissues. Representative images of DHX9 expression are shown in Figure 7C. A Spearman correlation analysis revealed a significant correlation between the expression levels of MCM2 and DHX9 (*R* = 0.195, *P* = 0.027), as well as between the levels of MCM3 and DHX9 (*R* = 0.248, *P* = 0.005) in osteosarcoma tissues. Taken together, MCM2 and MCM3 were correlated with DHX9 in tumor samples and were associated with poor prognosis in osteosarcoma patients.

**Table 1:** MCM2 and MCM3 expression status n (%)

Clinicopathologic parameters	MCM2 expression level				*p* value	MCM3 expression level				*p* value
	Negative	Low	Middle	Strong		Negative	Low	Middle	Strong	
Gender					0.983					0.708
Male	24	17	19	20		22	18	21	19	
Female	16	10	12	11		15	14	9	11	
Age (years)					0.994					0.723
< 18	21	14	17	16		17	17	16	18	
≥ 18	19	13	14	15		20	15	14	12	
Location					0.565					0.487
Femur	22	11	13	20		18	15	19	14	
Tibia	11	9	10	6		8	11	8	9	
Elsewhere	7	7	8	5		11	6	3	7	
Tumor necrosis rate (%)					0.159					0.067
< 90	31	20	27	29		31	22	28	26	
≥ 90	9	7	4	2		6	10	2	4	
Cortical destruction					0.754					0.884
Yes	33	24	26	28		32	27	25	27	
No	7	3	5	3		5	5	5	3	
Recurrence					0.000*					0.000*
Yes	3	7	19	24		3	9	17	24	
No	37	20	12	7		34	23	13	6	
Metastasis					0.514					0.203
Yes	20	15	21	18		17	17	21	19	
No	20	12	10	13		20	15	9	11	
Enneking stage					0.000*					0.000*
I	38	25	21	18		34	30	23	15	
II	2	2	10	13		3	2	7	15	

*P < 0.05.

**Table 2:** Impact of prognostic factors on TFS and OS by univariate analysis in osteosarcoma

Clinicopathologicparameters	Tumor-free survival				Overall survival			
	NO.	HR	95% CI	*p*	NO.	HR	95% CI	*p*
Gender								
Male	80	0.702	0.394–1.251	0.231	80	0.795	0.434–1.457	0.457
Female	49				49			
Age (years)								
< 18	68	1.374	0.800–2.360	0.250	68	0.861	0.487–1.524	0.608
≥ 18	61				61			
Location								
Femur	66	1.189	0.856–1.651	0.302	66	1.034	0.718–1.491	0.856
Tibia	36				36			
Elsewhere	27				27			
Tumor necrosis rate (%)								
< 90	107	0.670	0.302–1.485	0.324	107	0.672	0.285–1.586	0.364
≥ 90	22				22			
Cortical destruction								
Yes	111	0.959	0.452–2.037	0.914	111	0.983	0.440–2.194	0.966
No	18				18			
MCM2								
0	40	2.393	1.812–3.160	0.000*	40	2.570	1.881–3.512	0.000*
1	27				21			
2	21				21			
3	31				31			
MCM3								
0	37	2.502	1.873–3.343	0.000*	37	2.582	1.893–3.523	0.000*
1	32				32			
2	30				30			
3	30				30			
Enneking stage								
I	102	3.361	1.927–5.862	0.000*	102	4.142	2.325–7.381	0.000*
II	27				27			

*P < 0.05.

**Table 3:** Variables predictive of survival by COX proportional hazards model in osteosarcoma

	Parametes	Wald χ^2^	Risk Ratio	95% CI	*P*
TFS	MCM2	4.333	1.603	1.028–2.499	0.037
	MCM3	4.939	1.696	1.064–2.703	0.026
OS	MCM2	4.568	1.690	1.045–2.734	0.033
	MCM3	4.757	1.718	1.056–2.795	0.029

## DISCUSSION

Currently, the 5-year survival rate for patients with osteosarcoma is about 70%. However, it has remained almost unchanged over the past three decades. Thus, it is necessary to explore new therapeutic targets. A SWATH™ experiment, a label-free quantitative proteomic approach, was used to identify osteosarcoma-related nuclear proteins. In this study, 62 upregulated nuclear proteins and 87 downregulated nuclear proteins were identified in osteosarcoma cells compared to osteoblast cells. Then, 23 nuclear proteins potentially associated with osteosarcoma tumorigenesis were selected for proteomic validation by qRT-PCR, and 12 nuclear proteins were verified by western blotting, many of which were reported closely related to tumorigenesis. Velmurugan *et al*. reported that ANP32A was highly expressed in oral squamous cell carcinoma and could act as a potential biomarker for the prognosis of oral cancer patients with lymph node metastasis [19]. CTBP1, a co-repressor of tumor suppressor genes, increases breast tumor growth *in vitro* and in preclinical orthotopic xenograft models [20]. FHL1 has a tumor-suppressive role in tongue squamous cell carcinoma and accordingly may be a useful target for gene therapy [21]. As Rb and IMPDH2 are related to the progression of osteosarcoma [22, 23], siRNA transfection and a CCK-8 assay were performed for the functional screening of the other 9 nuclear proteins. The results showed that knockdown of MCM2 or MCM3 inhibited osteosarcoma cell proliferation in MNNG/HOS cells, suggesting that they played important roles in the tumorigenesis of osteosarcoma.

Several studies have reported a role of MCM family proteins in carcinogenesis. Hua *et al*. reported that high mRNA expression levels of *MCM2* and *MCM3* were correlated with a poor outcome and thus might be clinically useful molecular prognostic markers in glioma [24]. Coincidentally, the expression levels of MCM2 and MCM3 are sensitive markers for predicting aggressive and recurrent behaviors of ameloblastoma [25]. The overexpression of MCM2 and MCM3 increased the proliferation and migration of medulloblastoma [26]. A MCM2-targeted strategy might be an effective therapy for the treatment of highly malignant breast cancers [27]. These findings indicate that *MCM2* and *MCM3* act as oncogenes in tumorigenesis. To date, the role and underlying mechanism of MCM2 and MCM3 involved in osteosarcoma remain largely unclear. To assess the effects of MCM2 and MCM3 on osteosarcoma cells, a CCK-8 assay and colony formation assay were performed. In agreement with previous studies, we found that the knockdown of MCM2 and MCM3 resulted in significant cell growth inhibition *in vitro*. To further clarify the roles of MCM2 and MCM3 in osteosarcoma proliferation, MNNG/HOS cells with the stable knockdown of MCM2 or MCM3 were injected into nude mice. The results showed that MCM2 or MCM3 knockdown could dramatically impede tumor growth *in vivo*.

Although several studies have reported the role of MCM2 and MCM3 in tumorigenesis, the underlying mechanisms are largely unknown. Costa *et al*. reported that GINS and Cdc45 activated Mcm2-7 to initiate eukaryotic DNA replication [28]. It has recently been shown that activated Notch downregulated MCM2 and MCM6 to regulate cell proliferation, differentiation, and apoptosis [29]. In the present study, the co-immunoprecipitation technique was used to explore the underlying mechanism by which MCM2 and MCM3 influence osteosarcoma. After mass spectrometry-based protein identification, several proteins attracted our attention, including DHX9, DDX21, RPL4, FBL, RPL7, and RPL7A. We pulled down MCM2 and MCM3 and probed their interactions with other proteins. We presented evidence to support interactions between MCM2/MCM3 and DHX9. No interaction was found between either MCM2 or MCM3 and other proteins. Additionally, MCM2 and MCM3 co-localized with DHX9. This study, for the first time, demonstrates the interactions of MCM2/MCM3 with DHX9 in osteosarcoma cells.

DHX9 has been implicated in tumor cell maintenance by participating in transcriptional and translational steps and DNA replication [30–32]. To evaluate if MCM2 and MCM3 functioned via DHX9, a rescue experiment was performed. The overexpression of DHX9 rescued the MCM2 and MCM3 knockdown-induced osteosarcoma growth inhibition, showing that the control of cell proliferation by MCM2 and MCM3 was largely dependent on DHX9. As reported previously, DHX9 interacted with EGFR to activate transcription of EGFR-responsive genes. EGFR was an oncogene overexpressed in several human cancers [33]. In addition, DHX9 was reported to be a NF-κB binding partner [34], which played an important role in tumorigenesis. Therefore, DHX9 regulates various oncogenes and biological processes in tumors. Overexpression of DHX9 rescued the MCM2 and MCM3 knockdown-induced osteosarcoma growth inhibition by regulating numerous biological processes. Additionally, our results showed that knockdown of DHX9 inhibited osteosarcoma cell proliferation. Further validation showed that high MCM2 and MCM3 expression levels in osteosarcoma were associated with a poor prognosis. In addition, positive correlations between both MCM2 and MCM3 and DHX9 were found in osteosarcoma.

Collectively, knockdown of MCM2 or MCM3 dramatically inhibits osteosarcoma cell growth *in vitro* and *in vivo*. More importantly, this report provides, for the first time, experimental evidence for interactions of MCM2 and MCM3 with DHX9 in osteosarcoma cells and the importance of these interactions, clarifying the molecular mechanisms of MCM2 or MCM3-mediated tumorigenesis. MCM2 and MCM3 may be sensitive biomarkers to predict prognosis for osteosarcoma patients. Therefore, intervention targeting the MCM2/3–DHX9 axis may be a feasible and effective strategy for the treatment of osteosarcoma.

## MATERIALS AND METHODS

### Cell lines and cell culture

One human osteoblast cell line (hFOB 1.19), three osteosarcoma cell lines (MNNG/HOS, MG63, and U2OS), and the 293T cell line were used. The cells were maintained at 37°C in a humidified atmosphere containing 5% CO_2_. They were cultured in Dulbecco's modified Eagle's medium (MNNG/HOS, MG63, and 293T) or RPMI-1640 (U2OS) supplemented with 10% fetal bovine serum (South America Origin; Biowest, Logan, UT, USA), 100 U/mL penicillin (Sigma-Aldrich, St. Louis, MO, USA) and 100 mg/mL streptomycin (Sigma-Aldrich). The HFOB 1.19 cell line was cultured according to ATCC protocols.

### SWATH™ measurement

Nuclear proteins were extracted using NE-PE Nuclear and Cytoplasmic Extraction Reagents (Thermo Fisher Scientific, Rockford, IL, USA) according to the manufacturer's protocol. Then, 100 μg of each sample was reduced with dithiothreitol (DTT) and alkylated with iodoacetic acid (IAA), followed by digestion overnight at 37°C with trypsin (Promega, Madison, WI, USA). The peptides were separated in 0.1% formic acid (FA) and eluted in a linear gradient of 5–30% solvent B (98% ACN, 2% H2O, and 0.1% FA) over 90 min at a flow rate of 300 nL/min. Samples were analyzed by mass spectrometry in two phases, data-dependent acquisition (DDA) and SWATH acquisition, on the same samples using the same gradient conditions. The library generation and SWATH™ data file processes were described previously [35]. Proteins with at least 2 peptides were used for relative quantitation analyses. With four technical replicates for each sample, the relative quantitation analysis was performed using a *t-test* to determine if group means were significantly different given the standard deviation and the number of samples. Differences in protein levels with a *P-value* of less than 0.05 were considered significant in our experiment.

### RNA isolation and quantitative real-time PCR assays

Total RNA was extracted with TRIzol reagent (Invitrogen, Carlsbad, CA, USA) and quantified using the NanoDrop 2000 (Thermo Fisher Scientific, Waltham, MA, USA) according to the manufacturers’ protocol. First-strand cDNA was synthesized using the PrimeScript RT Reagent Kit (TaKaRa, Shiga, Japan). RT-PCR was performed with SYBR Green Premix Ex Taq (TaKaRa). All reactions were performed in a 10-μL reaction volume in triplicate. The expression levels of genes were measured using the comparative Ct method. The primer sequences are described in Supplementary Table 1.

### Western blot analysis

Lysates were extracted using a mixture of T-PER Protein Extraction Reagent (Thermo Fisher Scientific), PhosSTOP (Roche, Basel, Switzerland), and Complete Mini (Roche). The protein samples were separated by 6% or 8% sodium dodecyl sulfate–polyacrylamide gel electrophoresis and were transferred to nitrocellulose membranes (Millipore, Billerica, MA, USA). After blocking in 5% non-fat milk, the membranes were incubated with primary antibodies, which are described in Supplementary Table 2. The secondary antibody was anti-rabbit IgG (Sigma-Aldrich, 1:5000) or anti-mouse IgG (Sigma-Aldrich, 1:5000). Subsequent visualization was performed using SuperSignal West Femto Maximum Sensitivity Substrate (Thermo Fisher Scientific).

### Oligonucleotide transfection and stable cell line generation

Pre-designed siRNA (Ribobio, Guangzhou, China) and plasmids (Public Protein/Plasmid Library, Nanjing, China) were transfected into cells using Lipofectamine 2000 Reagent (Invitrogen) following the manufacturer's protocol. For assays of proliferation, cell cycle, migration, and invasion, and for RNA extraction and western blotting, cells were used 48 h after transfection. To construct a stable cell line, scramble control, MCM2-specific shRNA, or MCM3-specific shRNA lentiviral particles (Biotend, Shanghai, China) were infected into cells according to the manufacturer's protocol.

### Cell proliferation and colony formation assays

To examine cell proliferation, 48 h after transfection, 3000 cells were seeded into each well of a 96-well plate and incubated. A 10-μL aliquot of CCK-8 (Dojindo, Kumamoto, Japan) was added to triplicate wells and incubated for 2 h. Absorbance at 450 nm was measured. Each measurement was performed in triplicate, and the experiments were repeated twice. For the colony formation assay, 48 h after siRNA transfection, 1 × 10^3^ MNNG/HOS or U2OS cells were seeded in 6-well plates. After 10 days, cells in each well were fixed with 100% methanol for 30 min and stained with 0.1% crystal violet for 30 min. Finally, cell colonies were counted. The assays were independently conducted three times.

### Animal experiments

All animal experiments were approved by the Ethics Committee of the Shanghai Jiao Tong University Affiliated Sixth People's Hospital. For tumor growth assays, MNNG/HOS cells stably expressing sh-control, sh-MCM2, or sh-MCM3 were injected subcutaneously into the left scapulas of nude mice (6-week-old BALB/c-nu/nu, 6 per group, 2 × 10^6^ cells per mouse). The tumor volume was monitored twice a week and was calculated using the formula: V = 0.5 × length × width^2^. After 6 weeks, the mice were euthanized. For the histological analysis, primary tumors were harvested at necropsy and fixed in 10% formalin. The fixed samples were then embedded in paraffin and three non-sequential serial sections were obtained for each animal.

### Co-immunoprecipitation and mass spectrometry protein identification

Co-immunoprecipitation was performed using 293T, MNNG/HOS, and U2OS cells. Equal amounts of protein (3000 μg) were incubated with IgG, MCM2, or MCM3 antibodies at 4°C overnight and then the mixtures were incubated with protein A/G magnetic beads at 4°C for 3 h. The beads were washed using phosphate-buffered saline containing 1% Triton X-100 and eluted using 2× protein loading buffer at 100°C for 10 min. IgG-, MCM2-, or MCM3-bound proteins were resolved by SDS–PAGE and stained using the Silver Staining Kit (Beyotime Biotechnology, Shanghai, China). For the mass spectrometry analysis, MCM2-bound or MCM3-bound proteins were resolved by SDS–PAGE and stained with Coomassie blue R250 (Solarbio, Beijing, China). After destaining, reduction, and trypsin digestion for 12 h, the peptides were extracted using acetonitrile. The peptides were analyzed using a NanoLC System (NanoLC-2D Ultra; Eksigent, Dublin, CA, USA) equipped with a Triple TOF 5600 mass spectrometer (AB SCIEX, Framingham, MA, USA). Protein identification was performed using ProteinPilot4.1 (AB SCIEX). For this study, a strict unused confidence cutoff of >1.3 and ≥2 peptides were used for protein identification.

### Confocal immunofluorescence

Confocal immunofluorescence was performed using MNNG cells. Briefly, cells were fixed and incubated with rabbit polyclonal anti-MCM2 or anti-MCM3 antibody and mouse monoclonal anti-DHX9 antibody at 4°C overnight and then incubated with secondary antibodies (Invitrogen) for 1 h. Finally, the cells were incubated with DAPI (1:1000) for 5 min and viewed with a Fluoview FV1000 microscope (Olympus, Tokyo, Japan).

### Clinical samples and immunohistochemistry (IHC)

A total of 129 patients diagnosed with osteosarcoma were treated at the Shanghai Sixth People's Hospital. They received primary surgical treatment and preoperative and postoperative neoadjuvant therapy. The median age was 18 years old (range: 5–84 years). There are 80 males and 49 females. The recurrence rate is 41.09%. There are 66 cases located in the femur, 36 cases located in the tibia and 27 cases located in other bones. The follow-up period ranged from 24 to 64 months, and the median time was 36 months. Ethics approval was obtained from the local hospital ethic committees and written consent was obtained from each patient before sample collection. A standard IHC staining procedure was followed. Paraffin-embedded sections were cut at 4 μm, dewaxed in xylene, and treated by microwaving at 60°C for 20 min in EDTA buffer (pH 9.0) for antigen retrieval. Each slide was blocked for endogenous peroxidase activity by incubation in 0.3% H_2_O_2_ for 10 min, and then incubated at 37°C with a 1:100 dilution of the polyclonal antibody against MCM2 (Bioworld, Nanjing, China), MCM3 (1:300, Bioworld), DHX9 (1:200, Proteintech), or Ki-67 (1:200, DAKO). Slides were then rinsed three times in PBS, incubated for 30 min using the EnVision Staining Kit (DAKO, USA), and washed three times in PBS. Color development was performed for 3–10 min in a moist chamber at room temperature using DAB. Slides were counterstained in hematoxylin and dehydrated in graded ethyl alcohol (70%, 90%, and 100%). The primary antibody was replaced with PBS in sections used as negative controls. Assessment of IHC staining was carried out independently by two expert pathologists. Any discordance was solved by discussion until consensus was reached. The IHC signal intensities were scored as 0 (negative), 1 (low), 2 (middle), or 3 (strong). For ki-67, the percentage of ki-67-positive cells was calculated.

### Statistical evaluation

The data were compiled and analyzed using SPSS version 21.0. Comparisons between different groups were performed using Chi-squared tests. The independent prognostic significance of the parameters was estimated using the Cox proportional hazards model. Survival curves were generated using the Kaplan–Meier method. The correlations between MCM2, MCM3, and DHX9 were analyzed using the Spearman correlation test. The tumor-free survival time was defined as the period from surgery to the presence of new local lesions. The overall survival time was defined as the length of time between surgery and death. Results were considered significant at *P* < 0.05.

## SUPPLEMENTARY MATERIALS FIGURES AND TABLES



## Abbreviations

MCM2: Minichromosome maintenance protein 2
MCM3: Minichromosome maintenance protein 3
DHX9: DExH-box helicase 9
NUPR1: Nuclear Protein 1
DIA: data-independent acquisition
TFS: tumor-free survival
OS: overall survival
GO: gene ontology
KEGG: Kyoto Encyclopedia of Genes and Genomes
