# Supplementary material for: Minichromosome maintenance protein 2 and 3 promote osteosarcoma progression via DHX9 and predict poor patient prognosis
Source: Oncotarget. 2017 Feb 18;8(16):26380–93. doi: 10.18632/oncotarget.15474 (PMC5432265; doi:10.18632/oncotarget.15474)
Supplement: Supplementary file 1 [file oncotarget-08-26380-s001.pdf]

Minichromosome maintenance protein 2 and 3 promote osteosarcoma progression via DHX9 and predict poor patient prognosis

Supplementary Materials

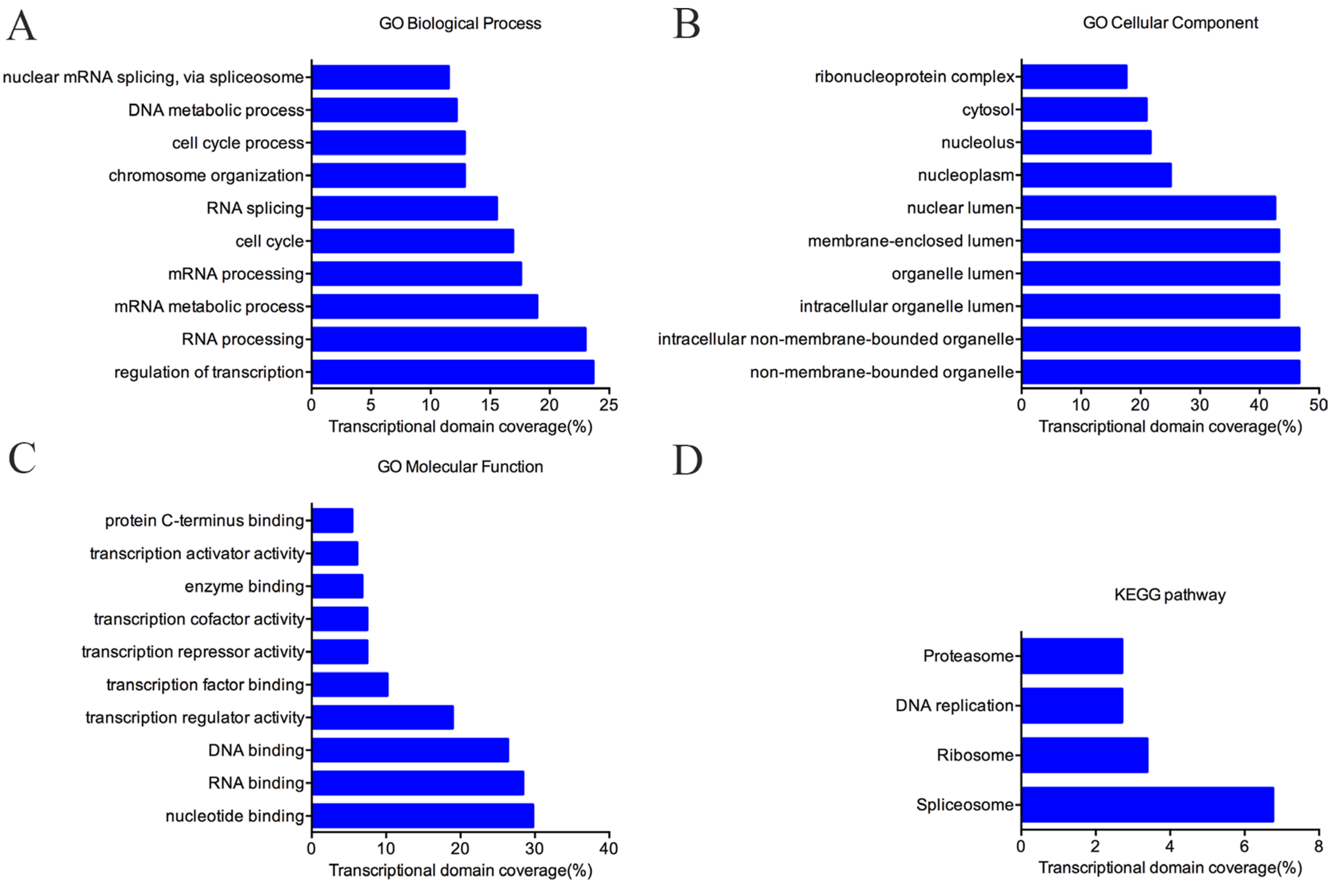

Supplementary Figure 1: Summaries of the GO and KEGG pathway analyses of dysregulated nuclear proteins in osteosarcoma cell lines.

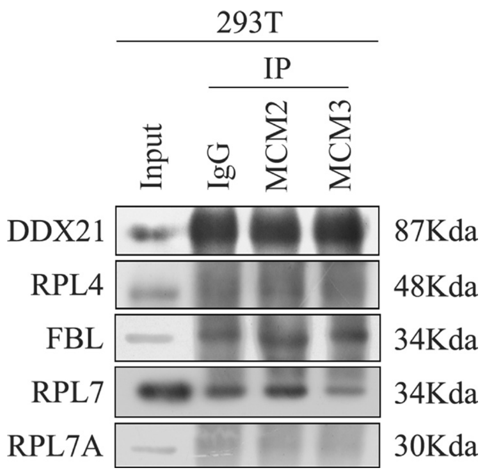

Supplementary Figure 2: Whole-cell lysates were immunoprecipitated with the anti-MCM2 antibody or anti-MCM3 antibody followed by immunoblotting with anti-DDX21, RPL4, FBL, RPL7, and RPL7A antibodies in the 293T cell line.

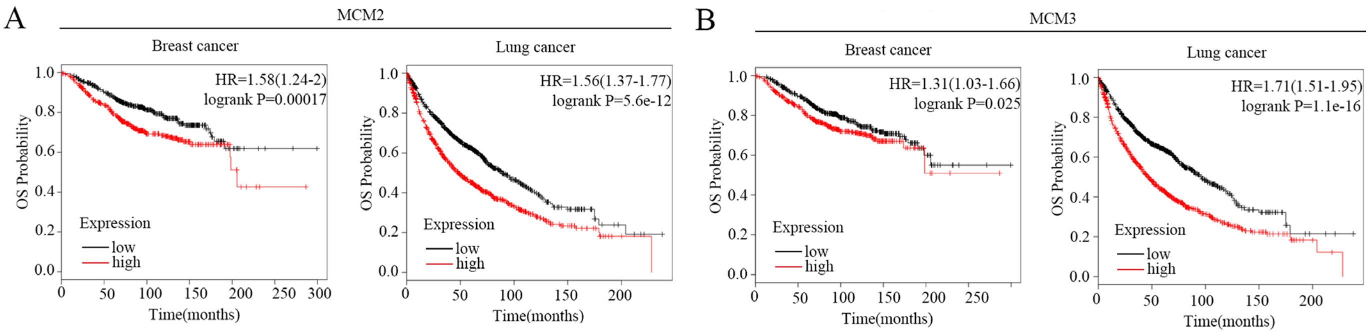

Supplementary Figure 3: Kaplan–Meier survival analyses were performed using microarray data (http://www. kmplot. com) for breast cancer and lung cancer patients.

Supplementary Table 1: Primer sequences used in the manuscript

Gene name		Sequence (5′->3′)
SUB1	Forward	CGTCACTTCCGGTTCTCTGT
	Reverse	TGATTTAGGCATCGCTTCGC
ANLN	Forward	TGCACCATTGGCACAAACAG
	Reverse	CCAGATTCAGCTCGAGGGAC
CSRP1	Forward	AGGCCTCTTGCCTTTGAGTC
	Reverse	GGATCCTCCCATCAAGCAGG
IFI16	Forward	AGAAGTGCCAGCGTAACTCC
	Reverse	TTGTGGTCAGTCGTCCATGC
PKM	Forward	CACCATGCGTGTTGTTCCTG
	Reverse	GGTGTCCCAACCTACCAGTG
MCM3	Forward	ACCCTGTCTACGGCAGGTAT
	Reverse	GTAACGGTGCATCCGAAGGA
MCM2	Forward	TCCGAGTGCTTTGTCTCCAC
	Reverse	AGCAGCCTGAATACGCAACT
CTBP1	Forward	GATGAGACAAGGGGCCTTCC
	Reverse	GAGGTTGGGTGCATCCTTCA
ANP32A	Forward	TTGAGTCCAACAGGCCATTGA
	Reverse	GATACACCACTGAGTCTTGCT
ANP32E	Forward	GTGAGCCCTTTTGTCAGGCT
	Reverse	TCCTTTTGAGAGAGCGGGAG
NME1	Forward	TTTGCCGGCCTGGTGAAATA
	Reverse	GAAGTCTCCACGGATGGTCC
PTMS	Forward	ATGTCCTGCCCCATCCCTAT
	Reverse	ACAGAAGCGCGACCATTTCT
IMPDH2	Forward	TTGGAGGCAATGTGGTCACT
	Reverse	GCCAGCACTTCCTGCGTAA
RB1	Forward	ACCTCACATTCCTCGAAGCC
	Reverse	TTGTTGGTGTTGGCAGACCT
GTF3C4	Forward	ACCAGTCCTGCCAGAGTTTG
	Reverse	AGGGGCTTTGCAGTAACCTC
FHL1	Forward	GTGAATTTCTGTCCGGCTGC
	Reverse	GTGTGTCTACACGGTGGGTC
TATDN1	Forward	GTGGCAATAGGAGAATGCGG
	Reverse	CTCCCCCTACACACCGATCT
NME2	Forward	GACCGACCATTCTTCCCTGG
	Reverse	TGATGTTCCTGCCAACCTGA
PFDN4	Forward	TCCAAGAGGACGGAATGTGGA
	Reverse	TTCTGCAGCCGCCTTCTTCAT
PFDN5	Forward	TTGTCCACGTCCATTGCTCA
	Reverse	ATTCTTTCCCCTCGTTGCTCT
SSU72	Forward	GTAGGGTGGGAACCCAAGCG
	Reverse	CCGTTTGCTGAGGATGTTGTG
RBBP7	Forward	CGCCGCGGCTCTTTCT
	Reverse	TCAAACACTGTTCTAAGATGACGAC
ZYX	Forward	CAGAACCAAAACCAGGTGCG
	Reverse	CAGCCACATTCTGCCTCTGA
DHX9	Forward	GGAAGCGAAGGCTGATCTGA
	Reverse	TGGTGGAATTTCCCATGCCA

Supplementary Table 2: Primary antibodies for western blot in the manuscript

Protein name	Dilution ratio	Reagent brand
Rb	1:500	Bioworld Technology
MCM2	1:1000	Cell Signaling Technology
GTF3C4	1:500	Proteintech
MCM3	1:1000	Cell Signaling Technology
PKM	1:500	Bioworld Technology
IMPDH2	1:500	Bioworld Technology
CTBP1	1:500	Bioworld Technology
ANP32A	1:500	Bioworld Technology
FHL1	1:500	Proteintech
NME1	1:500	Bioworld Technology
NME2	1:500	Bioworld Technology
CSRP1	1:500	Bioworld Technology
DHX9	1:1000	Proteintech
DDX21	1:1000	Proteintech
RPL4	1:500	Proteintech
FBL	1:500	BBI Life Sciences
RPL7	1:500	BBI Life Sciences
RPL7A	1:500	Proteintech
β-actin	1:20000	Sigma-Aldrich
